# Foundations of Human Consciousness: Imaging the Twilight Zone

**DOI:** 10.1523/JNEUROSCI.0775-20.2020

**Published:** 2021-02-24

**Authors:** Annalotta Scheinin, Oskari Kantonen, Michael Alkire, Jaakko Långsjö, Roosa E. Kallionpää, Kaike Kaisti, Linda Radek, Jarkko Johansson, Nils Sandman, Mikko Nyman, Mika Scheinin, Tero Vahlberg, Antti Revonsuo, Katja Valli, Harry Scheinin

**Affiliations:** ^1^Turku PET Centre, University of Turku and Turku University Hospital, FI-20521 Turku, Finland; ^2^Department of Perioperative Services, Intensive Care and Pain Medicine, Turku University Hospital, FI-20521 Turku, Finland; ^3^University of California, Irvine, Irvine, California 92868; ^4^Department of Intensive Care, Tampere University Hospital, FI-33521 Tampere, Finland; ^5^Department of Psychology and Speech-Language Pathology, and Turku Brain and Mind Center, University of Turku, FI-20014 Turun yliopisto, Finland; ^6^Department of Anesthesiology and Intensive Care, Oulu University Hospital, FI-90029 Oulu, Finland; ^7^Department of Radiation Sciences, Umeå University, 901 87 Umeå, Sweden; ^8^Department of Radiology, Turku University Hospital, FI-0521 Turku, Finland; ^9^Institute of Biomedicine and Unit of Clinical Pharmacology, University of Turku and Turku University Hospital, FI-20014 Turun yliopisto, Finland; ^10^Institute of Clinical Medicine, Biostatistics, University of Turku and Turku University Hospital, FI-20014 Turun yliopisto, Finland; ^11^Department of Cognitive Neuroscience and Philosophy, School of Bioscience, University of Skövde, 541 28 Skövde, Sweden

**Keywords:** anesthesia mechanisms, consciousness, dexmedetomidine, positron emission tomography, propofol, sleep

## Abstract

What happens in the brain when conscious awareness of the surrounding world fades? We manipulated consciousness in two experiments in a group of healthy males and measured brain activity with positron emission tomography. Measurements were made during wakefulness, escalating and constant levels of two anesthetic agents (experiment 1, *n* = 39), and during sleep-deprived wakefulness and non-rapid eye movement sleep (experiment 2, *n* = 37). In experiment 1, the subjects were randomized to receive either propofol or dexmedetomidine until unresponsiveness. In both experiments, forced awakenings were applied to achieve rapid recovery from an unresponsive to a responsive state, followed by immediate and detailed interviews of subjective experiences during the preceding unresponsive condition. Unresponsiveness rarely denoted unconsciousness, as the majority of the subjects had internally generated experiences. Unresponsive anesthetic states and verified sleep stages, where a subsequent report of mental content included no signs of awareness of the surrounding world, indicated a disconnected state. Functional brain imaging comparing responsive and connected versus unresponsive and disconnected states of consciousness during constant anesthetic exposure revealed that activity of the thalamus, cingulate cortices, and angular gyri are fundamental for human consciousness. These brain structures were affected independent from the pharmacologic agent, drug concentration, and direction of change in the state of consciousness. Analogous findings were obtained when consciousness was regulated by physiological sleep. State-specific findings were distinct and separable from the overall effects of the interventions, which included widespread depression of brain activity across cortical areas. These findings identify a central core brain network critical for human consciousness.

**SIGNIFICANCE STATEMENT** Trying to understand the biological basis of human consciousness is currently one of the greatest challenges of neuroscience. While the loss and return of consciousness regulated by anesthetic drugs and physiological sleep are used as model systems in experimental studies on consciousness, previous research results have been confounded by drug effects, by confusing behavioral “unresponsiveness” and internally generated consciousness, and by comparing brain activity levels across states that differ in several other respects than only consciousness. Here, we present carefully designed studies that overcome many previous confounders and for the first time reveal the neural mechanisms underlying human consciousness and its disconnection from behavioral responsiveness, both during anesthesia and during normal sleep, and in the same study subjects.

## Introduction

Experimental anesthesia and natural sleep are powerful research tools in the study of human consciousness (Fiset et al., 1999; Alkire et al., 2000; Horovitz et al., 2009; Boveroux et al., 2010; Långsjö et al., 2012; Liu et al., 2013; Akeju et al., 2014; Warnaby et al., 2016). Neural correlates of consciousness are often claimed to be found by comparing brain activity data collected during two states: wakefulness and a presumed unconscious state. This paradigm is, however, controversial in two fundamental ways. First, the state of consciousness is often defined by behavior (i.e., unconsciousness by lack of meaningful responses to external stimuli). Unresponsiveness (UR) does not, however, ensure unawareness (Owen et al., 2006; Huang et al., 2018) or the absence of internally generated experiences (Brice et al., 1970; Radek et al., 2018) and is, thus, by definition, not unconsciousness. Indeed, a conscious state can be defined as having experiences, also referred to as contents of consciousness. Yet, experimental studies rarely characterize the explored states explicitly or beyond behavioral properties (Bonhomme et al., 2019). In a connected state, such as during normal wakefulness, the contents of consciousness are modulated by incoming sensory information, resulting in conscious awareness of actual physical stimuli. In a disconnected state, the contents of consciousness are seldom related to incoming sensory information and typically consist of only internally generated experiences. Unconsciousness (i.e., the absence of experiences) also represents a disconnected state. Table 1 summarizes the characteristics of these conditions (modified from Sanders et al., 2012; Bonhomme et al., 2019), clarifying the multidimensional nature of human consciousness. Importantly, a disconnected state should be viewed as characteristic for successful general anesthesia, and complete unconsciousness is difficult to confirm in experimental settings.

**Table 1.** Cognitive and behavioral characteristics of connected consciousness, disconnected consciousness and unconsciousness

	Connected consciousness	Disconnectedness	
		Disconnected consciousness	Unconsciousness
Awareness of external stimuli	Yes	No	No
Behavioral responsiveness	Yes^a^	No	No
Subjective experiences	Yes	Yes	No

^a^Responsiveness may be absent in rare cases such as the locked-in syndrome or during muscle paralysis in conjunction with unsuccessful general anesthesia. Modified from Sanders et al. (2012) and Bonhomme et al. (2019).

The second problem concerning experimental anesthesia as a proxy to explore consciousness is the assumption that differences between wakefulness and (presumed) unconsciousness would straightforwardly reflect the neural correlates of consciousness (Scheinin et al., 2018b). This is not the case, as anesthetic drugs have sedative and other direct and indirect effects on the brain, which may affect the interpretations of the obtained data. Pharmacologic limitations can be resolved, e.g., by exploring physiological sleep), but drowsiness and sleep pressure also affect brain activity independent of major changes in the state of consciousness. In the current study, we aimed to separate changes in brain activity related specifically to consciousness from the overall effects of anesthesia and sleep. We applied novel experimental approaches to tackle previous limitations and to address the following three main questions: (1) what are the neural correlates of connected consciousness, as assessed by identifying the specific differences in brain activity between connected and disconnected states of consciousness; (2) are anesthesia and physiologic sleep similar or different in this respect; and (3) are the brain areas affected by transitions from connected to disconnected states and from disconnected to connected states the same or different? We used positron emission tomography (PET) imaging to measure brain activity, reflected by changes in regional cerebral blood flow (rCBF) in two separate experiments in the same group of healthy subjects. Measurements were made during wakefulness, stepwise escalating and constant levels of two anesthetic agents (experiment 1), and during sleep-deprived wakefulness and non-rapid eye movement (NREM) sleep stages (experiment 2).

In both experiments, the following two sets of analyses were conducted: the first aimed to discover the overall effects of anesthesia and sleep by comparing different doses of the drugs and different sleep stages to awake baseline values. The second aimed to identify state-specific patterns in brain activity. Here, only within-subject connected and disconnected states of consciousness, with minimal confounding effects, were compared. Maintained responsiveness to external auditory stimuli and an awake sleep-deprived state indicated a connected state. Unresponsive anesthetic states and verified sleep stages, where a subsequent immediate report of mental content included no signs of awareness of the surrounding world (see Materials and Methods), indicated a disconnected state.

## Materials and Methods

### 

#### 

##### Subjects

The study was approved by the Ethics Committee of the Hospital District of Southwest Finland and the Finnish Medicines Agency Fimea, and was registered at ClinicalTrials.gov (identifier NCT01889004). Altogether, 40 20- to 30-year-old healthy, American Society of Anesthesiologists (ASA) 1 (according to the ASA physical status classification system), right-handed male volunteers were recruited. Only male subjects were included because of the radiation exposure related to PET imaging. All subjects were interviewed and thoroughly examined by a licensed physician (A.S.). A standard 12-lead electrocardiogram (EKG) was administered, and blood and urine samples were analyzed to confirm the subjects' health status. Exclusion criteria included any somatic illness, regular medication or drug allergy, history of any psychiatric disorder or substance abuse, cardiac arrhythmias, hearing impairment, propensity to severe nausea in connection to anesthesia, blood donation in the preceding 90 d, prior participation in a PET/ single photon emission computed tomography study, any contraindication to magnetic resonance imaging (MRI), detected unsuitability based on initial electrophysiological measurements, detected unsuitability based on anatomic MRI scans, and pathologic findings in laboratory tests or positive urine drug screen results. All subjects provided written informed consent according to the Declaration of Helsinki.

##### Experimental designs and study objectives

Our aim was to investigate human consciousness in two separate experiments, using PET imaging, with two different anesthetic agents and physiological sleep. Functional brain imaging data were obtained during escalating and constant levels of anesthesia, in different states of consciousness (responsive and connected vs unresponsive and disconnected) and in different sleep stages. Scans were compared within and between subjects to identify brain regions fundamental for the regulation of human consciousness. Our experimental designs tried to bypass some previous limitations related to drug administration and heterogeneous dosing schemes. Specifically, we eliminated the confounding sedative and possible other drug-induced effects as well as the confounding effect of sleep pressure on brain activity. We also extended the assessment of the state of consciousness beyond behavior and conducted interviews to verify the phenomenal state of the subjects (i.e., the presence or absence of experiences during unresponsiveness).

**Figure 1.**
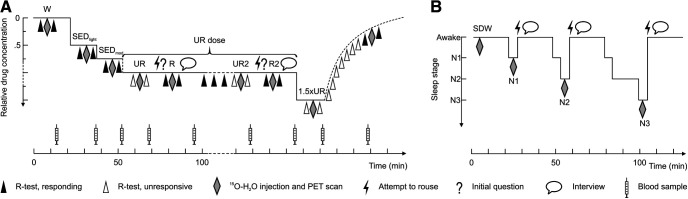
Design of Experiments 1 and 2. Behavioral states of interest in the anesthesia study are abbreviated as follows: W, wakeful baseline; SED_light_, light sedation; SED_mod_, moderate sedation state. Note the fixed-dose anesthetic level (UR dose) with steady-state infusion in UR, R, UR2, and R2. For details, see Materials and Methods. Behavioral states of interest in the sleep study are abbreviated as follows: SDW, sleep-deprived wakefulness; N1, N2, N3, NREM sleep stages N1, N2 and N3. For details, see Materials and Methods.

Subjects were investigated during drug-induced anesthesia (exposure to either propofol or dexmedetomidine) and during physiological sleep. In both experiments, brain activity was measured using functional PET imaging of rCBF, using ^15^O-labeled H_2_O as a tracer. In experiment 1 (*n* = 39), scans were obtained during escalating and constant anesthetic levels, which represented different states of consciousness driven by forced awakenings from an unresponsive state. In experiment 2 (*n* = 37), the same subjects were studied on average 18 weeks later, and brain activity, reflected by changes in rCBF, was measured during sleep deprivation and NREM sleep stages N1, N2, and N3. Experiment 1 (anesthesia) was open and randomized. Permuted blocks were applied to achieve balanced groups across treatments. Detailed study outlines of both experiments are described in the subsections Anesthesia study (experiment 1) and Sleep study (experiment 2), and are schematically illustrated in Figure 1.

##### Anesthesia study (experiment 1)

The subjects abstained from the use of alcohol and any medication for at least 48 h and fasted overnight before the experiment. Two forearm veins were cannulated for the administration of study drugs and the PET radiotracer and for blood sampling. Intravenous anesthetics, propofol (10 mg/ml; Propofol Lipuro, B. Braun) or dexmedetomidine (100 μg/ml; Dexdor, Orion Pharma) were administered using target-controlled infusions (TCIs) with previously described pharmacokinetic parameters (Marsh et al., 1991; Talke et al., 2003). A Harvard 22 syringe pump (Harvard Apparatus) and a portable computer running Stanpump software was used for drug administration (by Dr. Steven L. Shafer; www.opentci.org/code/stanpump). Plasma targets were used. Electroencephalogram (EEG) was recorded with a 64-channel Ag/AgCl active electrode cap (EasyCap) with electrodes placed according to the 10–10 system and with NeurOne 1.3.1.26 software (Mega Electronics), and Tesla #MRI 2013011 and #MRI 2013012 amplifiers (Mega Electronics). Additionally, two pairs of bipolar electrodes were used to monitor the electro-oculogram and EKG.

The level of consciousness was manipulated with either propofol (*n* = 19, one subject withdrew after randomization) or dexmedetomidine (*n* = 20) using TCI with stepwise increasing drug concentrations. Predefined concentrations for loss of responsiveness (LOR) from a preceding dose-finding study in the same subjects were used as reference (Kallionpää et al., 2018; Scheinin et al., 2018a). The initial target concentration of the infusion depended on the individually determined concentrations, starting from a 0.5× LOR concentration for each subject, then 0.75× LOR – 1.0× LOR until UR was reached. UR was defined as a participant's inability to respond to a standardized prerecorded responsiveness test (see below). If UR was not reached with 1.0× LOR, additional 0.25× increments compared with the previous target level were applied at ∼13 min intervals until UR was reached in every subject. “Moderate sedation” was defined as the last responsive anesthetic level before UR, and “light sedation” as the preceding responsive anesthetic level. The concentration needed to induce UR determined the anesthetic level, which was maintained as a pseudo-steady-state infusion using TCI for at least 13 min. Then, an attempt was made to arouse the subject with verbal stimuli (subject addressed by name) and, if necessary, mild tactile stimuli (a shake in the shoulder). In case of successful recovery to a responsive state (R), structured interviews to probe the subjects' experiences from the UR period were conducted (for details, see subsection Assessment of the state of consciousness). The subjects were then left unstimulated and a second UR (UR2) was targeted without adjustment of drug exposure. Thereafter, a second awakening and interview were conducted (R2). Thus, two cycles of different states of consciousness (responsive–unresponsive) were attempted during a constant-rate anesthetic drug infusion. After UR2, or if awakening on the first or second round was unsuccessful, or if a subject did not achieve the UR2 state, the drug concentration was increased by 50% to achieve a deeper level of anesthesia (1.5× UR). Finally, the drug infusion was terminated, and the subjects were allowed to recover. At baseline, at sedative levels and at each achieved state thereafter, brain activity changes reflected by rCBF were measured with repeated PET scans (for details, see subsection Positron emission tomography imaging).

The behavioral state of the subjects was classified based on a responsiveness test (i.e., R test) that was presented through headphones. The R test consisted of a prerecorded set of 10 sentences with a semantically congruent (*n* = 5) or incongruent (*n* = 5) last word. The R test was played at every drug concentration level and whenever another constant-rate UR or R state was targeted. The subjects were instructed to respond by left or right handle-press according to the congruency of the sentence; allocation of hands corresponding to congruous sentences (left or right) was balanced. UR was defined as 0 of 10 handle-presses. Each R test block lasted ∼90 s, and the same sentence was never repeated. The R test was presented with the Presentation 17.0 stimulus delivery and experimental control software system (Neurobehavioral Systems). All instructions and stimuli were delivered via headphones. Detailed information regarding stimulus preparation has been described in our previous publication (Kallionpää et al., 2018).

##### Sleep study (experiment 2)

Thirty-seven subjects from experiment 1 participated in experiment 2 (another 2 subjects withdrew after the anesthesia study). Consumption of alcohol and medications was not allowed in the preceding 48 h and intake of caffeine-containing products was prohibited for 16 h before the study session. The likelihood of falling asleep while inside the PET scanner was increased by requiring sleep deprivation for at least 30 h before the imaging session. EEG equipment similar to that used in experiment 1 was used to record EEG and to monitor sleep stages during the PET scan. For complete polysomnography (PSG), two additional bipolar electrodes were attached on the mentalis and submentalis muscles to record electromyogram. EKG was monitored as in experiment 1.

Sleep staging to determine PET scan onsets was done by visual inspection of online PSG by an experienced sleep technician (K.V.) according to the 2013 Academy of American Sleep Medicine (AASM) *Manual for the Scoring of Sleep and Associated Events* guidelines. The aim was to first scan each subject in the awake state (sleep-deprived wakefulness) and then in as many different sleep stages as possible. The maximum number of scans was restricted to five to avoid excessive radiation exposure. After the first scan during sleep-deprived wakefulness, the subjects were allowed to fall asleep. Once the subject fell asleep, a second PET scan was immediately started (NREM stage N1), followed by a third scan during light sleep (NREM stage N2) and a fourth scan during deep sleep (NREM stage N3). After each scan, the subjects were awakened and interviewed in detail for mental content during the verified sleep stage (for details, see the subsection Assessments of the state of consciousness). Final sleep staging was conducted offline by two experienced sleep technicians for the 90 s scan time that was used for PET data analysis, applying the 2013 AASM guidelines, with an inter-rater agreement of 93.1% (Cohen's κ = 0.908, *p* < 0.001).

##### Assessment of the state of consciousness

Maintained responsiveness always indicated a connected state. In both experiments, reports were collected to probe subjective experiences during the preceding unresponsive anesthetic or NREM sleep conditions. In experiment 1, the subjects were asked an initial question after each evoked awakening whether dreaming had been present during the unresponsive period (answer options: “yes,” “no,” “uncertain”). Thereafter, a PET scan was performed to attain an immediate scan from the evoked awakening. A more detailed interview followed, requesting the subjects to report any subjective experiences they might have had during the unresponsive period, including possible awareness of the study surroundings (Radek et al., 2018). In experiment 2, the detailed interview was conducted immediately after the awakening.

The interviews were digitally recorded and later transcribed word by word for systematic content analysis conducted by two independent judges to verify disconnectedness during the unresponsive periods. The answer to the initial question in experiment 1 (yes, no, uncertain) was analyzed to assess the presence of subjective experiences. In content analysis from both experiments, the judges divided the interview reports into the following three main categories: (1) reports including no recall of any subjective experiences, (2) white reports (i.e., reports where the participant had a strong impression of experiences during unresponsiveness, but could not recall any specific content), and (3) reports including specific content. The reports including specific content were further categorized as either including internally or externally generated experiences. Internally generated experiences involved hallucinatory contents of consciousness, either dreaming or memory incorporation of the research environment (i.e., experiences related to things/persons that were present or events that had occurred before unresponsiveness ensued), while externally generated experiences referred to awareness of the current environment (experiences related to verifiable stimuli that the participant could not have expected to occur during the experimental session). Reports of no recall of any experiences, white reports, and reports including internally generated experiences were considered to verify disconnectedness, whereas reports of awareness were considered as signs of connectedness during unresponsiveness.

##### Magnetic resonance imaging

For each subject, an anatomic brain MRI scan [T1 3D, T2 axial, FLAIR (fluid-attenuated inversion recovery) coronal] was obtained before experiment 1 for subsequent image preprocessing and exclusion of any brain anomalies. A Philips Ingenuity PET-MR 3 T scanner (Philips Medical Systems) was used. A trained neuroradiologist (M.N.) evaluated the anatomic images for any pathologic findings. Isotropic T1 3D was also used as an anatomic reference in PET data analysis.

##### Positron emission tomography imaging

PET imaging was performed using an ECAT HRRT (high-resolution research tomograph) brain scanner (Siemens CTI). The HRRT is a dual-layer, LSO (lutetium oxyortho-silicate)/LYSO (lutetium yttrium oxyortho-silicate) crystal detector scanner characterized by a nearly isotropic 2.5 mm intrinsic spatial resolution. In the reconstructed images, spatial resolution varies from 2.5 to 3 mm in the radial and tangential directions and from 2.5 to 3.5 mm in the axial direction in the 10 cm field of view (FOV), and the total length of axial FOV is 250 mm, covering most of the brain. Subjects were positioned in the scanner in the supine position, using a standard headrest and a Velcro band over the forehead to minimize head movements, and head motion was monitored with a high-precision, stereotaxic tracking device (Polaris Vicra, Northern Digital) attached to the subject's head.

To assess rCBF, [^15^O]O_2_ was produced with a low-energy deuteron accelerator Cyclone 3 (IBA) at Turku University Hospital. The target gas with [^15^O]O_2_ was mixed with pure H_2_ to produce water vapor in a hot (700°C) quartz furnace. Radiopharmaceutical grade [^15^O]H_2_O was produced according to good manufacturing practices using an automated Radiowater Generator (Hidex). A 300 MBq dose of [^15^O]H_2_O was administered in 15 s by an automated infusion system (Rad Injector, Tema Sinergie). Emission data in list-mode format were recorded over the duration of the [^15^O]H_2_O administration and the subsequent 120 s. Point of departure (POD) for emission data were determined offline as the time point where the “trues” count rate exceeded the “randoms” count rate. By default, the list-mode data were histogrammed in two (60 and 30 s) 3D sinograms from POD onward. In case the external motion recordings indicated significant (>2.5 mm) within-frame motion, subframes were formed until subthreshold level motion was assured (Johansson et al., 2016). In most cases, subframing was not needed; yet, 91 subframes in 31 (of 302) sessions were generated for experiment 1, and 10 subframes in 6 (of 116) sessions were generated for experiment 2, and some subframes were discarded (in 29 sessions in experiment 1 and in 2 sessions in experiment 2) because of shortage of data. There were no marked differences in the number of incidences between the two drugs in experiment 1. Transmission data acquired just before the first [^15^O]H_2_O administration were used to generate photon attenuation maps, while a single-scatter simulation algorithm was used to estimate the proportion of scattered events and randoms were estimated from the block singles. All corrections were included in an iterative image reconstruction procedure including resolution modeling (point spread function-ordinary Poisson-ordered subsets expectation-maximization; 12 iterations, 16 subsets; Comtat et al., 2008) and motion compensation of the attenuation maps (Johansson et al., 2016). Motion compensated framewise data were summed to form a 90 s sum image for subsequent analysis.

##### Drug concentration measurements

Blood samples for drug concentration measurements were drawn into EDTA tubes from a cannulated forearm vein in experiment 1. Samples were drawn at baseline and at the end of each drug target infusion step. Additionally, a sample was taken in each behavioral state (i.e., whenever the state of consciousness was presumed to have changed). Concentrations of dexmedetomidine in plasma were measured with HPLC with tandem mass spectrometry. Propofol concentrations were measured with HPLC and fluorescence detection (Yeganeh and Ramzan, 1997).

##### Neuroimaging data analysis and statistical considerations

Image preprocessing was performed with standard PET techniques, as described above, and an average image of the summed PET images was formed for each condition for each subject. Across-subject image alignment, registration, and normalization were performed using statistical parametric mapping software (SPM8 and 12, Wellcome Institute). A reference frame from the baseline scan was used as a target to obtain initial between-sessions realignment and motion correction. The mean PET image was coregistered with the skull-stripped anatomic MRI and the session images were resliced accordingly into MRI voxel size (1 × 1 × 1 mm). Nonlinear mapping from the MRI to the MNI standard space was estimated using unified segmentation in SPM8, and the deformations were subsequently applied to the MRI and coregistered PET images. All normalized PET images were smoothed using an isotropic Gaussian kernel of 12 mm FWHM. Proportional scaling was used in the PET analyses.

Partial least-squares (PLS) software was used to analyze the data for rCBF pattern changes over state transitions. PLS is a multivariate statistical analysis technique that analyzes associations between two sets of data. Here we used PLS to identify brain activity patterns that differ between experimental conditions. The PLS output consists of a set of latent variables (LVs), which are linear combinations of initial variables that maximally covary with the corresponding conditions. The statistical significance of each LV was calculated with permutation tests. To assess the reliability of voxels contributing to the LV, bootstrapping was used. The bootstrap ratio is the ratio of the weights to the SEs estimated from bootstrapping. Therefore, the larger the magnitude of a bootstrap ratio, the larger is the weight (i.e., contribution to the latent variable) and the smaller the SE (i.e., higher stability; McIntosh and Lobaugh, 2004; Mišić et al., 2016).

Five thousand permutations were computed to determine the significance of each LV, and 5000 bootstrap iterations were run to assess the reliability of identified saliences. Voxels with saliences >2.575 × their SE, corresponding to approximately *p* < 0.01, were considered statistically significant. All comparisons yielded two LVs of which LV1 explained 100% of the cross-block covariance and was significant with *p* < 0.001, while LV2, representing the residuals, was not significant. All figures are bootstrap ratio figures with thresholds of *p* < 0.01 for voxels significantly contributing to the pattern. Since PLS analyzes the data in a multivariate fashion, there is thus only one statistical test and no need to correct for multiple comparisons.

First, we conducted a mean-centered task PLS analysis to establish the patterns of relative blood flow changes between the activity seen in the normal wakeful state (baseline acquired in experiment 1 for all subjects) and the gradually deepening levels of anesthesia (dexmedetomidine or propofol) and sleep, using separate pairwise analyses. Next, we targeted an analysis to seek for patterns of altered brain activity specifically related to changes in the state of consciousness (connected vs disconnected). We used the same method to analyze state transitions within subjects, between connected and disconnected conditions under light dexmedetomidine or propofol anesthesia and natural sleep, while minimizing the confounding drug and sleep pressure effects. To achieve this, comparisons were now made during constant-dose anesthesia or between sleep-deprived baseline and N2 sleep. Successful scans for comparisons were obtained from 19, 14, and 9 subjects, respectively, in the propofol, dexmedetomidine, and sleep groups (“becoming disconnected”), and from 9 and 16 subjects, respectively, in the propofol and dexmedetomidine groups (“becoming connected”). Since only 2 of 13 previously awakened propofol subjects achieved an UR2, we used the condition with the least confounding drug effect (i.e., “moderate sedation” versus UR) to examine brain activity changes related to transition from a responsive (and connected) to an unresponsive (and disconnected) conscious state in the propofol group. The final number of successful comparisons between connected and disconnected states was dependent on obtaining both connected and disconnected scans from each subject, and the applied comparisons are clarified in the legends of Figure 3 and Extended Data Fig. 3-1.

The normality of variables was checked using the Shapiro–Wilk test. The Fisher's exact test was used to compare arousability and responsiveness between the treatments. Paired and unpaired *t* tests were used to compare measured drug concentrations between the disconnected and connected conditions.

## Results

### Realization of experimental designs

All of the targeted states, interviews, and scans were not obtained in every subject. In experiment 1, 13 of 19 propofol subjects (68%) and 16 of 20 dexmedetomidine subjects (80%) were arousable during the fixed-dose drug infusion (Fisher's exact test, *p* = 0.480, df = 1). No significant within-subject differences were observed in drug concentrations between the fixed-dose responsive and fixed-dose unresponsive states (*p* > 0.2 for both drugs; Table 2). The measured drug concentrations were higher in those subjects who were not arousable in both drug groups (*p* < 0.05 for both). The numbers of successful rCBF PET scans were [*n* = propofol (obtained from percentage of subjects), *n* = dexmedetomidine (obtained from percentage of subjects)] as follows: wakeful baseline [*n* = 19 (100%), *n* = 20 (100%)]; light sedation [*n* = 14 (74%), *n* = 6 (30%)]; moderate sedation [*n* = 19 (100%), *n* = 20 (100%)]; UR [*n* = 19 (100%), *n* = 20 (100%)]; R [*n* = 9 (47%), *n* = 16 (80%)]; UR2 [*n* = 2 (11%), *n* = 15 (75%)]; R2 [*n* = 2 (11%), *n* = 14 (70%)]; and 1.5 × UR [*n* = 15 (79%), *n* = 16 (80%)]. Four awakened propofol subjects could not be scanned in the R state because of fluctuations in behavior (three subjects) or intravenous line malfunction (one subject). Within-subject pairs of images were used in the connected/disconnected analysis and hence, the number of comparisons in this analysis may be different from the total number of obtained scans.

**Table 2.** Targeted and measured drug concentrations during experiment 1

Drug	Light sedation		Moderate sedation		UR dose/disconnected		UR dose/connected		1.5× UR dose		Recovery	
	Targeted	Measured	Targeted	Measured	Targeted	Measured	Targeted	Measured	Targeted	Measured	Estimated	Measured
**All subjects**												
Propofol (μg/ml)	1.13 (0.37)	0.73 (0.39)	1.37 (0.49)	1.01 (0.51)	1.78 (0.56)	1.48 (0.60)	1.47 (0.42)	1.13 (0.31)	2.74 (0.81)	2.46 (0.77)	1.14 (0.37)	1.16 (0.35)
	*n* = 13		*n* = 18		*n* = 18		*n* = 8		*n* = 15		*n* = 15	
Dexmedetomidine (ng/ml)	1.19 (0.38)	0.98 (0.54)	1.06 (0.54)	1.10 (0.58)	1.50 (0.56)	1.80 (0.66)	1.24 (0.33)	1.54 (0.37)	2.38 (1.05)	3.27 (1.32)	1.38 (0.51)	1.60 (0.64)
	*n* = 6		*n* = 20		*n* = 20		*n* = 16		*n* = 16		*n* = 17	
**Subjects who could be awakened during constant infusion**												
Propofol (μg/ml)					1.47 (0.42)	1.06 (0.25)	1.47 (0.42)	1.13 (0.31)*				
					*n* = 8		*n* = 8					
Dexmedetomidine (ng/ml)					1.24 (0.33)	1.48 (0.40)	1.24 (0.33)	1.54 (0.37)§				
					*n* = 16		*n* = 16					

Values are the mean (SD) targeted or estimated and measured drug concentrations in plasma during light and moderate (last responsive anesthetic level before losing responsiveness) sedation, disconnected and connected states of consciousness during constant infusion titrated to UR dose/disconnected and UR dose/connected, respectively, and deep unresponsive state (1.5× UR dose) and responsive state after terminating the drug infusion (recovery) in the propofol (*n* = 19) and dexmedetomidine (*n* = 20) groups. No statistically significant differences in the measured concentrations between the disconnected and connected states in subjects who could be awakened (*p = 0.880, df = 7; §*p* = 0.203, df = 15; paired *t* tests after Bonferroni correction). The numbers vary because not all states were achieved in every subject and because of a few missing blood samples. (Extended Data Figures 3-1, 3-2, 3-3, 3-4, 3-5, and 3-6).

In experiment 2, sleep-deprived baseline scans (awake) were not obtained from all subjects because of an inability to remain awake during the scan. Altogether, 32 subjects fell asleep at least once (86%). While some subjects reached the same sleep stage and were awakened from it several times; only the first successful scan obtained from each achieved sleep stage was used. The numbers of first successful rCBF PET scans were [*n* = state (achieved by % of subjects)]: sleep-deprived wakefulness [*n* = 22 (59%)]; N1 [*n* = 14 (38%]; N2 [*n* = 24 (65%)]; and N3 [*n* = 14 (38%)]. Within-subject pairs of images were used in the connected/disconnected analysis and hence, the number of comparisons in this analysis may be different from the total number of obtained scans.

In those subjects who could be interviewed, subjective experiences (composed of white reports and reports including specific content) were reported in 80% and 71% of the interviews in experiments 1 and 2, respectively. Most often, internally generated dreaming or memory incorporation were described. In experiment 1, the recall rates of subjective experiences were equal (80% of interviews) in both drug groups. In experiment 2, subjective experiences were reported in 58%, 66%, and 83% of the N1, N2 and N3 interviews, respectively.

Signs of awareness were reported by one subject receiving propofol and one subject receiving dexmedetomidine, both after the second unresponsive period in experiment 1, and by one subject after N2 sleep in experiment 2. The scans obtained from these states were not considered to represent a disconnected state and were excluded from the connected versus disconnected comparisons. Apart from these cases, unresponsiveness denoted disconnected, albeit mostly not unconscious, states.

### Separation of changes in brain activity related specifically to consciousness from the overall effects of anesthesia

**Figure 2.**
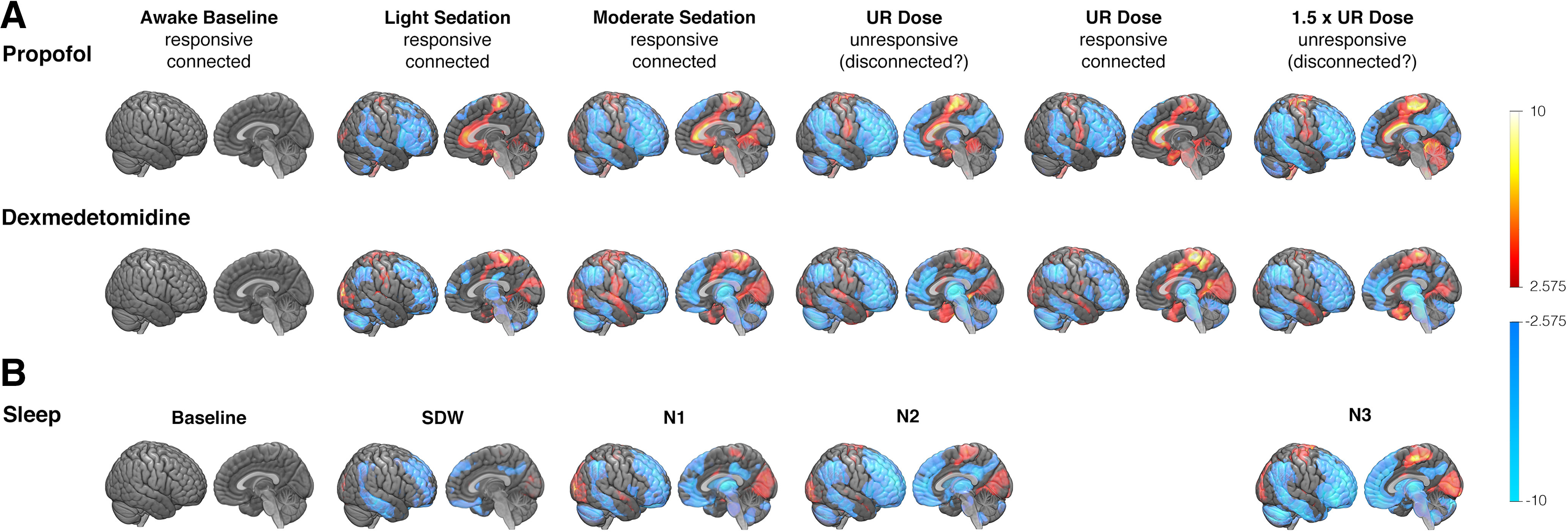
Relative rCBF suppression at different anesthetic levels, sleep stages, and behavioral states. ***A***, ***B***, Images showing the global pattern of rCBF changes in association with different levels of propofol or dexmedetomidine (***A***) and different sleep stages (***B***). All states are compared with a non-sleep-deprived awake baseline with no drug. Cool colors show the largest relative suppression and warm colors the smallest relative suppression (*p* < 0.01; color bars depict bootstrap ratios in PLS). Light and moderate sedation indicate responsive levels during escalating drug exposure. UR dose refers to drug concentration titrated individually to induce unresponsiveness, and 1.5× UR dose refers to 50% higher doses. The states of consciousness (connected or disconnected) during UR and 1.5× UR levels could not be verified because of a lack of immediate interviews in unarousable subjects and/or after terminating the infusion, and are therefore marked as “(disconnected?)”. Maximal suppression is seen in frontal and parietal cortical areas as well as in subcortical structures, and the pattern is evident already during light sedation, resembling the awake sleep-deprived state. The intensity of suppression increases with drug dose level and depth of sleep, regardless of the behavioral state. Light sedation (SED_light_; propofol, *n* = 14; dexmedetomidine, *n* = 6), moderate sedation (SED_mod_; propofol, *n* = 19; dexmedetomidine, *n* = 20), UR dose and UR (propofol, *n* = 19; dexmedetomidine, *n* = 20), UR dose and R forced awakening during anesthetic infusion (propofol, *n* = 9; dexmedetomidine, *n* = 16), 1.5× UR dose (propofol, *n* = 15; dexmedetomidine, *n* = 16); sleep-deprived wakefulness (SDW; *n* = 22); NREM sleep stages N1 (*n* = 14), N2 (*n* = 24), and N3 (*n* = 14); all targeted states were not achieved in all subjects.

In experiment 1, we scanned 39 healthy subjects with PET in multiple conditions varying in terms of administered anesthetic agent, the level of drug exposure and the subjects' responsiveness. All drug concentration levels and behavioral states were first compared with an awake baseline without drug to reveal the overall effects of the drugs on brain activity. We discovered that both drugs similarly suppressed rCBF. The most profound reductions were seen in frontal, parietal, and temporal cortical regions and subcortically mainly in the thalamus, whereas primary sensory and motor cortices were less affected (Fig. 2*A*). The portrayed effects were not indicative of behavior (responsiveness) or state of consciousness as they were already evident at sedative drug concentrations. They thus depicted the combined influences of the drug and the state of consciousness.

**Figure 3.**
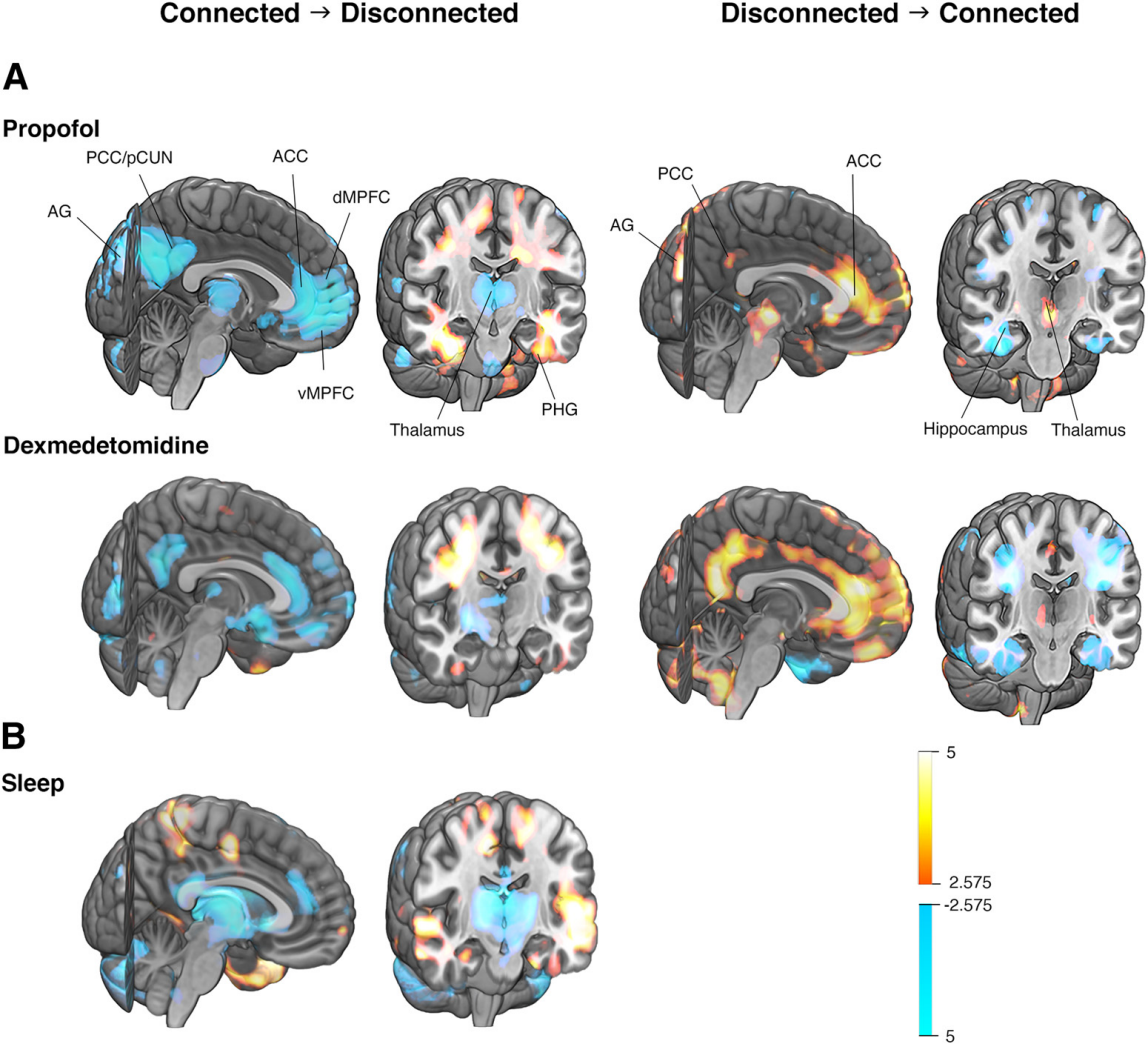
Differences in relative rCBF between connected and disconnected states of consciousness. A central core network of consciousness was revealed by imaging anesthetic-induced and sleep-induced state transitions. Cool colors show the largest relative suppression on becoming disconnected and warm colors the smallest (left); and warm colors show the largest relative activation on becoming connected and cool colors the smallest (right; *p* < 0.01, corrected; color bars depict bootstrap ratios in PLS). ***A***, During infusions of both propofol (top) and dexmedetomidine (middle), state-specific analyses between connected and disconnected conditions revealed that a network of core midline structures was activated and deactivated in a reciprocal manner, with minimal effects seen on the cortical surface. Activity of the thalamus, anterior and posterior cingulate cortices, precuneal area, and bilateral angular gyri showed the most consistent associations with the subject's state of consciousness. ***B***, During physiological sleep (bottom), transition from sleep-deprived wakefulness to N2 sleep revealed the deactivation of the same core structures. Again, changes in cortical surfaces were inconsistent. Brain regions with statistically significant differences are listed in Extended Data Figures 3-2, 3-3, 3-4, 3-5, and 3-6, and cortical renderings are shown in Extended Data Figure 3-1. AG, Angular gyrus; dMPFC, dorsomedial prefrontal cortex; pCUN, precuneus; PHG, parahippocampal gyrus; vMPFC, ventromedial prefrontal cortex. Successful scans for within-subject comparisons were compared in 19 (SED_mod_ → UR), 14 (R → UR2), and 9 (SDW → N2) propofol, dexmedetomidine, and sleep subjects (left: connected → disconnected), and in 9 (UR → R) and 16 (UR → R) propofol and dexmedetomidine subjects (right: disconnected → connected), respectively.

To unmask the confounding pharmacologic effects, we used the forced awakening paradigm during constant-rate anesthetic drug infusions and compared changes in brain activity between connected and disconnected states of consciousness at similar measured drug concentrations (Table 2). Most subjects were arousable during the fixed-dose infusions and 80% of the arousable subjects reported subjective experiences in the immediate interview. Thus, the subjects were mostly in a disconnected and conscious, rather than an unconscious, state. Within-subject comparisons were made between connected and disconnected states, where the concentration-dependent drug effects on the brain were controlled by the study design. We thereby explored (1) which functional changes best associate with the loss and return of connected consciousness and (2) whether the transitions between these two states of consciousness are reciprocal and symmetrical. We discovered that the activity of a restricted network of core midline brain structures including the thalamus, anterior cingulate cortex (ACC), posterior cingulate cortex (PCC), and the angular gyri in the inferior parietal lobules were consistently associated with the connected state (Fig. 3*A*, Extended Data Figs. 3-2, 3-3, 3-4, and 3-5). This network was activated and deactivated in an opposite (reversed) manner, independently of the drug administered. The more extensive suppression of frontoparietal cortical areas that was seen in comparisons with no-drug awake baseline was not manifested during transition to a disconnected state, nor was an analogous activation of these areas seen at recovery to a connected state. Some cortical effects were observed, but they were heterogeneous in terms of the direction of change, drug, and areas affected (Extended Data Fig. 3-1*A*). Consistent state-specific differences in brain activity were witnessed only within a restricted network of midline structures and the angular gyri.

10.1523/JNEUROSCI.0775-20.2020.f3-1 Extended Data Figure 3-1 Differences in relative rCBF on the cortical surface between connected and disconnected states of consciousness. Cortical renderings illustrating state-related changes in brain activity revealed by imaging anesthetic- and sleep-induced state transitions. Cool colors show the most relative suppression on becoming disconnected and warm colors the least (first, third, and fifth rows); and warm colors show the most relative activation on becoming connected and cool colors the least (second and fourth rows; *p* < 0.01, corrected; the color bar depicts bootstrap ratios in PLS). The figure illustrates minimal cortical effects, and they were heterogeneous in terms of the direction of change, drug, and areas affected. For subcortical renderings, see Figure 3. Successful scans for within-subject comparisons were compared in 19 (SED_mod_ → UR), 14 (R → UR2), and 9 (SDW → N2) propofol, dexmedetomidine, and sleep subjects (connected → disconnected; rows 1, 3, and 5) and in 9 (UR → R) and 16 (UR → R) propofol and dexmedetomidine subjects (disconnected → connected; rows 2 and 4), respectively. Download Figure 3-1, TIF file.

10.1523/JNEUROSCI.0775-20.2020.f3-2 Extended Data Figure 3-2 Supplementary Figure 3-2. Download Figure 3-2, DOCX file

10.1523/JNEUROSCI.0775-20.2020.f3-3 Extended Data Figure 3-3 Supplementary Figure 3-3. Download Figure 3-3, DOCX file

10.1523/JNEUROSCI.0775-20.2020.f3-4 Extended Data Figure 3-4 Supplementary Figure 3-4. Download Figure 3-4, DOCX file

10.1523/JNEUROSCI.0775-20.2020.f3-5 Extended Data Figure 3-5 Supplementary Figure 3-5. Download Figure 3-5, DOCX file

### Physiologic sleep resembles anesthesia

The same subjects (*n* = 37 because of two withdrawals) participated in the sleep experiment, where no pharmacologic interventions were used to manipulate consciousness. Compared with awake baseline (acquired in experiment 1), the suppression of rCBF during sleep deprivation and N1, N2 and N3 sleep resembled the effects of increasing anesthetic exposure (Fig. 2*B*). The largest suppression of blood flow was observed in the higher-order frontal, parietal, and temporal cortical regions and in some subcortical structures, such as the thalamus, whereas activity was relatively preserved in lower-order somatosensory and motor cortical regions. Overall, physiological sleep seemed to suppress blood flow similarly to the two different anesthetic agents.

Next, we tested whether the effect of strong sleep pressure and resulting drowsiness because of sleep deprivation could be minimized by comparing the sleep-deprived (connected) state to N2 sleep (disconnected state). Overall, N2 sleep was followed by a report with subjective experiences in 66% of the immediate interviews. Thus, most subjects were in a disconnected, rather than a fully unconscious, state. Within-subject comparisons were made between connected and disconnected states, aiming to reveal which functional changes associate best with a loss of connected consciousness. The results were clearly distinct from those of the first analysis. A restricted network of core midline structures, including the thalamus, anterior and posterior cingulate cortices, bilateral angular gyri, dorsolateral prefrontal cortex, and right caudate nucleus, was consistently associated with the state of consciousness (Fig. 3*B*, Extended Data Fig. 3-6). Cortical renderings showed that a sleep-induced change in the state of consciousness was accompanied with only minimal activity changes on the cortical surface (Fig. 3-1*B*).

## Discussion

It has been widely accepted that a broad network of frontoparietal cortical areas contribute to consciousness and its contents (Baars et al., 2003). It is also indisputable that brain activity is globally reduced and that communication between different brain areas is disrupted during different unconscious states. The activity of distinct medial or lateral subsystems has been implicated in awareness, specifically awareness of self and the environment, respectively (Boly et al., 2008). It is not equally well characterized, what specifically accounts for loss or recovery of a connected state, and what the necessary preceding effects are that enable this transition. The complexity of these phenomena in the brain and the diverse experimental designs across studies have complicated the forming of a unified view on the neural correlates and mechanisms of consciousness, resulting in an understandable rivalry between different theories (Reardon, 2019).

We used an established PET method to monitor brain activity and used novel designs to overcome some past limitations related to experimental studies on human consciousness. Two experiments targeting unresponsive anesthetic states and physiological sleep revealed that the induced conditions represented mostly disconnected, rather than unconscious, states. We discovered that connected and disconnected states of consciousness were best differentiated by activity in the core midline structures of the brain, including the thalamus, cingulate cortices, and angular gyri. Only minimal and inconsistent differences on the cortical surface were witnessed between these two conditions, suggesting a lesser contribution of the outer cortex to the connected state per se.

In previous studies using anesthesia and sleep, distinct patterns of altered brain activity and/or connectivity during unresponsive states have been described (Braun et al., 1997; Kajimura et al., 1999; Boveroux et al., 2010; Liu et al., 2013; Akeju et al., 2014; Ranft et al., 2016; Warnaby et al., 2017). It has been shown that information transfer across frontoparietal cortical areas is disrupted during sleep and anesthesia (Massimini et al., 2005; Boly et al., 2012), but there is also strong evidence to support that thalamic activity is more crucial and critically involved in cortical regulation (Alkire et al., 2000; Xie et al., 2011; Långsjö et al., 2012; Baker et al., 2014). Involvement of the insula (Warnaby et al., 2016) and angular gyri (Legostaeva et al., 2019) have also been demonstrated. Anesthesia and sleep have also both been shown to disrupt thalamic connectivity to the higher-order cortex (Akeju et al., 2014; Guldenmund et al., 2017), while lower-order sensory circuits are less impacted (Boveroux et al., 2010; Liu et al., 2013), experimentally supporting “cognitive unbinding” as mechanistic for unconsciousness (Mashour, 2013). Our current findings neither contradict any previous work, nor do we question the suppression of any previously described neuronal circuit in conjunction with anesthesia or sleep. We highlight, however, the importance of relevant comparisons in experimental studies on consciousness; all changes in brain activity do not exclusively reflect changes in the state of consciousness. Indeed, we were able to partly overcome confounding effects by choosing the most relevant scan as the wakeful reference. With our approach, a shift between connected and disconnected states associated best with the changes within a restricted network of midline structures and the angular gyri. Interestingly, the method used to manipulate consciousness (i.e., anesthetic agent or physiological sleep) seemed to have a minimal effect on the results.

Our findings suggest that widespread cortical suppression is not sufficient, albeit perhaps necessary, for loss of connected consciousness. In our previous study (Långsjö et al., 2012), awakening during constant-dose dexmedetomidine administration was associated with activation of the ACC, thalamus, and the brainstem (i.e., phylogenetically old cortical regions and the arousal system). The emerged regions overlap with distinct neuronal networks implicated in human consciousness. The default mode network (DMN) is considered to be foundational for self-referential mentation whereas the executive control network (ECN) for externally guided awareness. The salience network (SN) is thought to play a role in coordinating between the DMN and ECN (Menon and Uddin, 2010; Demertzi et al., 2013). Unresponsive states of different etiologies have been shown to associate with the suppression or disruption of functional connectivity within these networks (Boveroux et al., 2010; Qin et al., 2015; Guldenmund et al., 2017; Huang et al., 2020), which has been corroborated by the findings of the current study. Interestingly, decreased rCBF or BOLD fMRI signal in DMN areas and thalamus can also be seen in a psychedelic state induced by psilocybin (Carhart-Harris et al., 2012) and in DMN areas during meditation (Brewer et al., 2011). Both psychedelic and meditative states have been associated with decreased sense of self. Indeed, general anesthesia has been characterized as “fragmentation of selfhood” (Sleigh et al., 2018). We are tempted to speculate that while the global state seemed most reliant on the activity of the thalamus, the DMN, and the SN, a disconnected state also needs a preceding deactivation of the cortex.

Surprisingly identical effects were induced by the different interventions, despite the distinct molecular mechanisms of action of the two drugs and the complex cascades of sleep regulation (Saper et al., 2005). Our findings suggest a partly unitary neural mechanism to operate behind the investigated conditions. Indeed, dexmedetomidine has been suggested to induce a state resembling physiological sleep, as assessed by both behavioral and electrophysiological features (Nelson et al., 2003; Huupponen et al., 2008), whereas propofol is considered different in this respect. Interestingly, forced awakening turned out to be feasible also in most of the propofol subjects, and this quality may be exploited in experimental studies on consciousness.

When relating our findings to theories of the neural mechanisms of consciousness, the distinction between the state of being conscious versus the specific contents of consciousness becomes relevant. As to the contents of consciousness, most theories place the neural correlates of consciousness in specific cortical networks; the Neural Global Workspace theory (Dehaene and Changeux, 2011) in long-range frontoparietal connections; the Recurrent Processing theory (Lamme, 2010) in local recurrent activities in the ventral occipitotemporal cortex and the Posterior Hot Zone theory (Koch et al., 2016) in posterior cortical areas, excluding the frontal cortex. As to the state of being conscious, which is necessary for any contents of consciousness to manifest, most theories (Llinás et al., 1998; Tononi and Edelman, 1998; Koch et al., 2016) emphasize subcortical and thalamocortical connections. Our findings reveal the necessary and minimally sufficient mechanisms for the connected state, supporting and refining the latter theories. As we did not investigate any specific contents of consciousness, our results cannot resolve the current controversy between the posterior versus frontoparietal theories of the contents of consciousness. However, our results clearly show that frontal and frontoparietal cortical areas were strongly affected already before a disconnected state was reached. Thus, they (or indeed any superficial cortical areas) do not seem to be necessary for the connected state as such, although they may be necessary for particular contents of consciousness.

Important methodological limitations related to the present study need to be addressed. Verification of the disconnected state was based on the subjects' responsiveness and reports of mental content, neither of which can indisputably verify a person's actual state of consciousness. A motor response to a presented stimulus is dependent on the type and salience of the chosen stimulus, as well as the complexity of the requested behavioral output. The superiority of any stimulus has not, to our knowledge, been characterized. Retrospective subjective reports, in contrast, are strongly dependent on memory. Internal conscious experiences are commonly reported after experimental and clinical anesthesia (Sanders et al., 2012; Gyulaházi et al., 2016; Cascella et al., 2017; Radek et al., 2018) and on awakening from all stages of sleep (Nielsen, 2000). However, the lack of a dream report does not unequivocally indicate unconsciousness (Windt et al., 2016); and the lack of an awareness report after anesthesia does not necessarily prove disconnectedness (Sanders et al., 2017). Delayed interviews must be especially considered unreliable because of amnesia caused by anesthetics or sleep (Schwartz and Maquet, 2002; Hudetz, 2008). Retrospective reports remain, however, the only way to access subjective experiences during an unresponsive state. In a recent study on dreaming, a similar awakening paradigm with immediate interviews was used. Based on individual EEG patterns, it was possible to predict with 87% total prediction accuracy across all states whether a subsequent report from NREM and REM sleep included dreaming (Siclari et al., 2017), providing important validation of the report-based state classification.

Our study had several strengths. We used identical dosing schemes for two very different anesthetic drugs resulting in similar behavioral end points. The same subjects participated in the subsequent sleep study. This enabled cross-study and within-subject comparisons using identical data acquisition and analysis procedures. We identified a network of core brain structures where activity was consistently associated with the state of consciousness (connected or disconnected). Anesthesia and sleep had state-specific effects that were distinct, reciprocal, and separable from their overall effects on brain activity. Stringent and accurate definitions of the explored states and their proper comparisons are of vital importance, as anesthetic-induced unresponsiveness and sleep rarely provide complete unconsciousness.
