# Supplementary figures and images for: Foundations of Human Consciousness: Imaging the Twilight Zone
Source: J Neurosci. 2021 Feb 24;41(8):1769–78. doi: 10.1523/JNEUROSCI.0775-20.2020 (PMC8115882; doi:10.1523/JNEUROSCI.0775-20.2020)

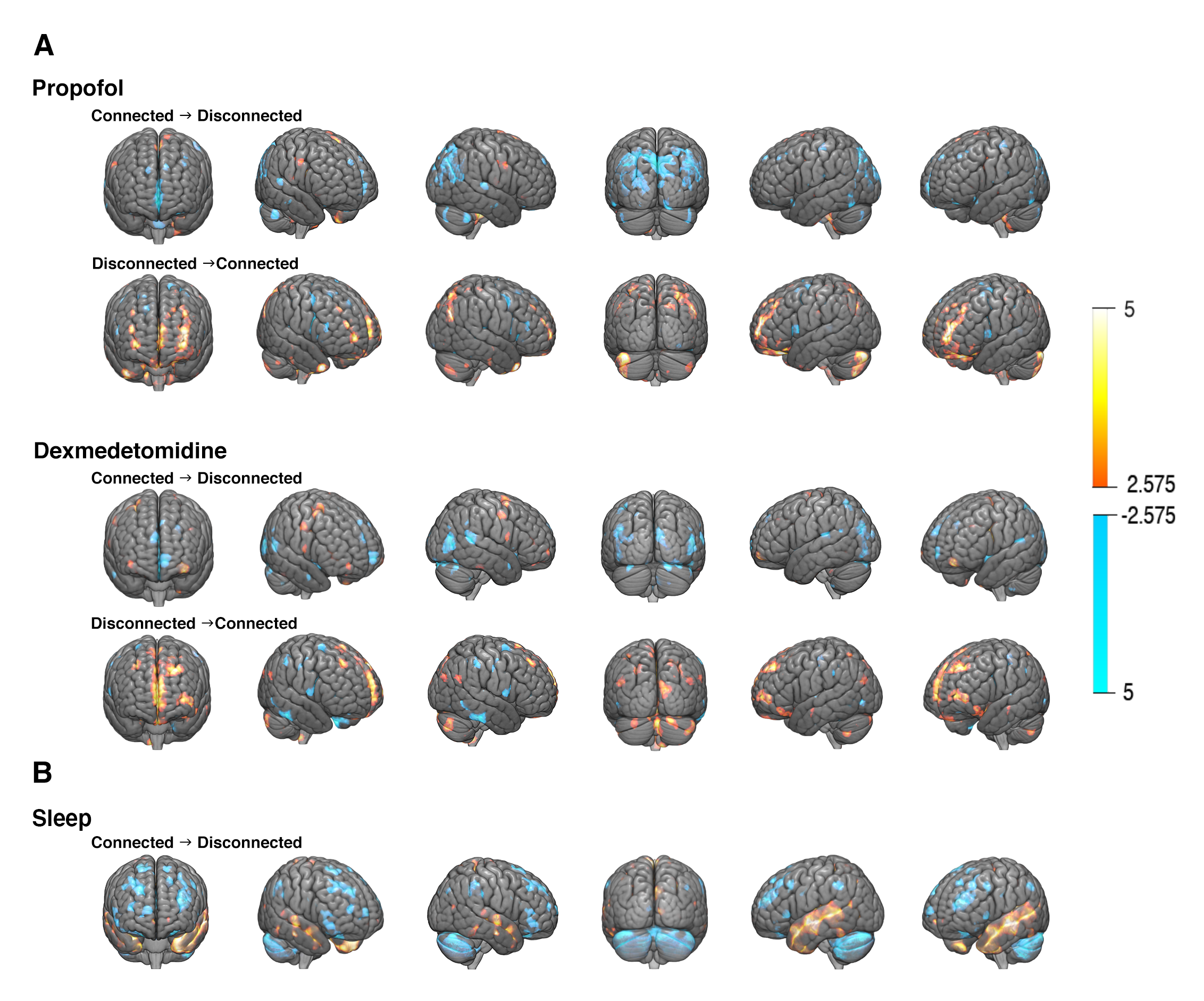

Supplement: Extended Data Figure 3-1 — Differences in relative rCBF on the cortical surface between connected and disconnected states of consciousness. Cortical renderings illustrating state-related changes in brain activity revealed by imaging anesthetic- and sleep-induced state transitions. Cool colors show the most relative suppression on becoming disconnected and warm colors the least (first, third, and fifth rows); and warm colors show the most relative activation on becoming connected and cool colors the least (second and fourth rows; p < 0.01, corrected; the color bar depicts bootstrap ratios in PLS). The figure illustrates minimal cortical effects, and they were heterogeneous in terms of the direction of change, drug, and areas affected. For subcortical renderings, see Figure 3. Successful scans for within-subject comparisons were compared in 19 (SEDmod → UR), 14 (R → UR2), and 9 (SDW → N2) propofol, dexmedetomidine, and sleep subjects (connected → disconnected; rows 1, 3, and 5) and in 9 (UR → R) and 16 (UR → R) propofol and dexmedetomidine subjects (disconnected → connected; rows 2 and 4), respectively. Download Figure 3-1, TIF file. [file ns-JN-RM-0775-20-s01.tif]
