# Supplementary material for: Foundations of Human Consciousness: Imaging the Twilight Zone
Source: J Neurosci. 2021 Feb 24;41(8):1769–78. doi: 10.1523/JNEUROSCI.0775-20.2020 (PMC8115882; doi:10.1523/JNEUROSCI.0775-20.2020)
Supplement: Extended Data Figure 3-2 — Supplementary Figure 3-2. Download Figure 3-2, DOCX file [file ns-JN-RM-0775-20-s02.docx]

**Figure 3-2.** Brain regions with statistically significant differences in relative regional cerebral blood flow between connected and disconnected states of consciousness during propofol infusion revealed by Partial least squares software.

| **Cluster Brain Regions** | **Peak Voxel MNI Coordinates (x,y,z)** | **Cluster Size (voxels)** | **BSR** | **p-value** |
| --- | --- | --- | --- | --- |
| **Negative Saliences** |  |  |  |  |
| R/L Anterior Cingulate Gyrus and R/L Ventromedial Prefrontal Cortex | 6 46 -2 | 3467 | -7.8543 | <0.0001 |
| R/L Posterior Cingulate Gyrus, R/L Precuneus and R/L Angular Gyrus | 6 -46 28 | 8693 | -7.4026 | <0.0001 |
| R Occipital Fusiform Gyrus | 16 -90 -16 | 333 | -5.2925 | <0.0001 |
| L/R Thalamus | -10 -24 8 | 1686 | -4.6389 | <0.0001 |
| R Superior Temporal Gyrus | 70 -22 10 | 53 | -4.5891 | <0.0001 |
| L Frontal Pole | -28 38 -20 | 178 | -4.4089 | <0.0001 |
| L Frontal Pole | -10 56 44 | 26 | -4.3288 | <0.0001 |
| R Cerebellum | 50 -48 -36 | 244 | -4.2327 | <0.0001 |
| L Inferior Temporal Gyrus | -60 -62 -18 | 193 | -4.1559 | <0.0001 |
| L Cerebellum | -40 -62 -40 | 382 | -4.149 | <0.0001 |
| L Occipital Fusiform Gyrus | -22 -88 -18 | 97 | -4.1477 | <0.0001 |
| L Lateral Inferior Occipital Cortex | -44 -88 4 | 126 | -4.14 | <0.0001 |
| L Postcentral Gyrus | -50 -20 62 | 73 | -4.0507 | 0.0001 |
| Pons | -4 -24 -46 | 371 | -3.9619 | 0.0001 |
| L Temporal Pole | -52 22 -14 | 32 | -3.6445 | 0.0003 |
| L Middle Frontal Gyrus | -34 40 44 | 23 | -3.6221 | 0.0003 |
| R Cerebellum | 46 -78 -36 | 127 | -3.2878 | 0.001 |
| L Middle Frontal Gyrus | -36 24 54 | 25 | -3.2517 | 0.0011 |
| R Cerebellum | 16 -78 -38 | 50 | -3.1934 | 0.0014 |
| R Frontal Orbital Cortex | 34 30 -16 | 60 | -3.1709 | 0.0015 |
| R Frontal Pole | 14 58 38 | 28 | -3.108 | 0.0019 |
| **Positive Saliences** |  |  |  |  |
| R Parahippocampal Gyrus and R Temporal Fusiform Cortex | 32 -16 -30 | 3504 | 7.9338 | <0.001 |
| R Postcentral Gyrus and R Precentral Gyrus | 34 16 28 | 16619 | 7.7469 | <0.0001 |
| L Precentral Gyrus and L Postcentral Gyrus | -32 0 -36 | 3052 | 5.8316 | <0.0001 |
| L Superior Frontal Gyrus | -2 20 66 | 191 | 4.6754 | <0.0001 |
| R Postcentral Gyrus | 64 -8 16 | 63 | 3.8299 | 0.0001 |
| L Supramarginal Gyrus | -56 -32 42 | 104 | 3.7433 | 0.0002 |
| L Cerebellum | -12 -70 -56 | 66 | 3.6024 | 0.0003 |
| R Inferior Frontal Gyrus, Pars Triangularis | 52 30 -6 | 22 | 2.9176 | 0.0035 |

Abbreviations: Montreal Neurological Institute (MNI), bootstrap ratio (BSR), right (R), left (L).
