# Supplementary material for: Foundations of Human Consciousness: Imaging the Twilight Zone
Source: J Neurosci. 2021 Feb 24;41(8):1769–78. doi: 10.1523/JNEUROSCI.0775-20.2020 (PMC8115882; doi:10.1523/JNEUROSCI.0775-20.2020)
Supplement: Extended Data Figure 3-3 — Supplementary Figure 3-3. Download Figure 3-3, DOCX file [file ns-JN-RM-0775-20-s03.docx]

**Figure 3-3.** Brain regions with statistically significant differences in relative regional cerebral blood flow between disconnected and connected states of consciousness during constant propofol infusion revealed by Partial least square software.

| **Cluster Brain Regions** | **Peak Voxel MNI Coordinates (x,y,z)** | **Cluster Size (voxels)** | **BSR** | **p-value** |
| --- | --- | --- | --- | --- |
| **Positive Saliences** |  |  |  |  |
| R Temporal Pole | 38, 16, -42 | 123 | 12.3852 | <0.0001 |
| R Angular Gyrus | 48, -70, 32 | 538 | 10.8938 | <0.0001 |
| R Posterior Cingulate Gyrus / Retrosplenial Cortex | 2, -44, 4 | 70 | 10.8411 | <0.0001 |
| L Frontal Pole, L/R Anterior Cingulate Gyrus, L/R Ventromedial Prefrontal Cortex | -34, 62, 12 | 7151 | 10.2789 | <0.0001 |
| L Anterior Parahippocampal Gyrus | -6, 8, -28 | 829 | 6.8136 | <0.0001 |
| R Frontal Pole | 34, 42, 44 | 31 | 6.4894 | <0.0001 |
| L Superior Parietal Lobe | -32, -62, 66 | 126 | 6.2663 | <0.0001 |
| L Frontal Pole | -36, 42, 40 | 70 | 5.996 | <0.0001 |
| R Cerebellum | 12, -76, -22 | 144 | 5.8779 | <0.0001 |
| L Cerebellum | -42, -80, -28 | 482 | 5.664 | <0.0001 |
| R Thalamus | 2, -20, -6 | 439 | 5.5605 | <0.0001 |
| L Angular Gyrus | -50, -74, 34 | 158 | 5.4981 | <0.0001 |
| R Frontal Pole | 36, 58, 22 | 46 | 5.2715 | <0.0001 |
| R Superior Parietal Lobe | 26, -60, 70 | 34 | 5.1292 | <0.0001 |
| Pons | 2, -16, -36 | 37 | 4.8282 | <0.0001 |
| L Angular Gyrus | -56, -54, 50 | 116 | 4.817 | <0.0001 |
| L Frontal Pole | -54, 38, -8 | 27 | 4.667 | <0.0001 |
| L Precuneus | -6, -72, 64 | 37 | 4.6446 | <0.0001 |
| R Precuneus | 6, -82, 48 | 49 | 4.418 | <0.0001 |
| R Supramarginal Gyrus | 64, -50, 36 | 46 | 4.4115 | <0.0001 |
| L Parahippocampal Gyrus | -20, 2, -12 | 83 | 4.3833 | <0.0001 |
| R Pallidum | 26, -18, 0 | 21 | 4.2162 | <0.0001 |
| R Cerebellum | 16, -42, -50 | 37 | 4.1972 | <0.0001 |
| L Supramarginal Gyrus | -68, -20, 30 | 25 | 4.0725 | <0.0001 |
| L Pallidum | -24, -14, 2 | 68 | 4.0681 | <0.0001 |
| R Posterior Cingulate Gyrus | 14, -42, 34 | 21 | 3.7995 | 0.0001 |
| R Lateral Occipital Cortex | 24, -88, 42 | 32 | 3.783 | 0.0002 |
| R Posterior Cingulate Gyrus | 2, -48, 28 | 64 | 3.613 | 0.0003 |
| R Frontal Orbital Cortex | 36, 16, -18 | 43 | 3.3934 | 0.0007 |
| L Temporal Pole | -48, 4, -38 | 23 | 3.3333 | 0.0009 |
| R Anterior Cingulate Gyrus | 10, -8, 36 | 22 | 3.3303 | 0.0009 |
| R Cerebellum | 54, -48, -36 | 24 | 3.2205 | 0.0013 |
| R Cerebellum | 16, -62, -46 | 44 | 3.2128 | 0.0013 |
| Brainstem/Pons | -14, -24, -24 | 32 | 3.0553 | 0.0022 |
| R Cerebellum | 48, -70, -44 | 59 | 2.8787 | 0.004 |
| **Negative Saliences** |  |  |  |  |
| L Supramarginal Gyrus | -56, -48, 26 | 124 | -11.1147 | <0.0001 |
| R Parahippocampal Gyrus | 30, -16, -28 | 2310 | -9.1862 | <0.0001 |
| R Occipital Pole and R Occipital Fusiform Gyrus | 32, -94, -10 | 459 | -9.0442 | <0.0001 |
| L Occipital Fusiform Gyrus | -32, -58, -20 | 1086 | -7.2767 | <0.0001 |
| R Superior Frontal Gyrus and R Middle Frontal Gyrus | 20, 18, 44 | 2002 | -5.6269 | <0.0001 |
| L Supramarginal Gyrus | -40, -40, 40 | 489 | -5.5524 | <0.0001 |
| L Central Opercular Cortex | -36, -6, 18 | 560 | -5.4647 | <0.0001 |
| L Middle Frontal Gyrus | -34, 8, 52 | 180 | -5.2084 | <0.0001 |
| L Superior Parietal Lobe | -14, -52, 62 | 95 | -5.1045 | <0.0001 |
| R Frontal Pole | 14, 44, 40 | 178 | -5.0014 | <0.0001 |
| L Lateral Inferior Occipoital Cortex | -44, -76, 8 | 141 | -4.9771 | <0.0001 |
| R Postcentral Gyrus | 54, -20, 24 | 236 | -4.913 | <0.0001 |
| L Superior Frontal Gyrus | -12, -6, 70 | 523 | -4.8561 | <0.0001 |
| R Precentral Gyrus | 30, -24, 64 | 23 | -4.7758 | <0.0001 |
| L Supramarginal Gyrus | -54, -26, 28 | 117 | -4.5675 | <0.0001 |
| R Supramarginal Gyrus | 50, -34, 38 | 152 | -4.5599 | <0.0001 |
| L Superior Parietal Lobe | -40, -46, 58 | 57 | -4.5376 | <0.0001 |
| R Precentral Gyrus | 36, -14, 48 | 90 | -4.2862 | <0.0001 |
| L Inferior Frontal Gyrus, Pars Opercularis | -54, 14, 10 | 162 | -4.2467 | <0.0001 |
| L Precentral Gyrus | -52, 2, 26 | 136 | -4.2251 | <0.0001 |
| L Superior Temporal Gyrus | -66, -34, 10 | 104 | -3.8392 | 0.0001 |
| R Superior Frontal Gyrus | 22, -8, 68 | 163 | -3.783 | 0.0002 |
| R Cerebellum | 32, -42, -52 | 29 | -3.6687 | 0.0002 |
| L Insular Cortex | -32, 22, 4 | 156 | -3.4898 | 0.0005 |
| R Superior Parietal Lobe | 22, -52, 56 | 53 | -3.4867 | 0.0005 |
| L Intracalcarine Cortex | -22, -70, 2 | 30 | -3.2738 | 0.0011 |
| L Occipital Fusiform Gyrus | -26, -88, -8 | 47 | -2.8505 | 0.0044 |

Abbreviations: Montreal Neurological Institute (MNI), bootstrap ratio (BSR), right (R), left (L).
