# Supplementary material for: Foundations of Human Consciousness: Imaging the Twilight Zone
Source: J Neurosci. 2021 Feb 24;41(8):1769–78. doi: 10.1523/JNEUROSCI.0775-20.2020 (PMC8115882; doi:10.1523/JNEUROSCI.0775-20.2020)
Supplement: Extended Data Figure 3-4 — Supplementary Figure 3-4. Download Figure 3-4, DOCX file [file ns-JN-RM-0775-20-s04.docx]

**Figure 3-4.** Brain regions with statistically significant differences in relative regional cerebral blood flow between disconnected and connected states of consciousness during constant dexmedetomidine infusion revealed by Partial least squares software.

| **Cluster Brain Regions** | **Peak Voxel MNI Coordinates (x,y,z)** | **Cluster Size (voxels)** | **BSR** | **p-value** |
| --- | --- | --- | --- | --- |
| **Positive Saliences** |  |  |  |  |
| L Nucleus Accumbens, R/L Anterior Cingulate Gyrus and R/L Ventromedial Prefrontal Cortex | -6 6 -8 | 6600 | 8.8685 | <0.0001 |
| R/L Posterior Cingulate Gyrus, R/L Precuneus and R Thalamus | 8 -56 18 | 3395 | 7.0568 | <0.0001 |
| L Frontal Pole | -24 50 42 | 823 | 5.9146 | <0.0001 |
| L Superior Frontal Gyrus | -2 20 72 | 271 | 5.8895 | <0.0001 |
| Cerebellum | 0 -80 -32 | 1977 | 5.3916 | <0.0001 |
| L Frontal Pole | -46 54 2 | 203 | 5.3682 | <0.0001 |
| R Superior Frontal Gyrus | 26 38 52 | 125 | 4.9191 | <0.0001 |
| L Angular Gyrus | -48 -72 24 | 218 | 4.7742 | <0.0001 |
| R Subthalamic Nucleus | 20 12 -28 | 117 | 4.6807 | <0.0001 |
| L Subthalamic Nucleus | -6 -12 -12 | 111 | 4.4123 | <0.0001 |
| R Cerebellum | 18 -90 -24 | 475 | 4.2522 | <0.0001 |
| R Caudal Superior Parietal Lobe | 30 -78 52 | 112 | 4.1143 | <0.0001 |
| L Cerebellum | -38 -48 -46 | 265 | 4.0629 | <0.0001 |
| L Caudal Superior Parietal Lobe | -28 -84 46 | 70 | 3.8677 | 0.0001 |
| R Occipital Pole | 10 -102 16 | 326 | 3.7994 | 0.0001 |
| R Cerebellum | 52 -72 -34 | 299 | 3.7608 | 0.0002 |
| L Superior Parietal Lobe | -38 -60 62 | 40 | 3.7119 | 0.0002 |
| L Thalamus | -16 -28 8 | 41 | 3.6683 | 0.0002 |
| L Cerebellum | -12 -42 -24 | 229 | 3.6085 | 0.0003 |
| R Cerebellum | 28 -44 -32 | 32 | 3.5521 | 0.0004 |
| Occipital Pole | 0 -90 2 | 70 | 3.5218 | 0.0004 |
| L Superior Parietal Lobe | -4 -70 68 | 116 | 3.5168 | 0.0004 |
| L Cerebellum | -30 -88 -30 | 132 | 3.4518 | 0.0006 |
| R Angular Gyrus | 44 -82 32 | 55 | 3.2926 | 0.001 |
| L Middle Frontal Gyrus | -30 24 60 | 23 | 3.2628 | 0.0011 |
| R Angular Gyrus | 62 -58 32 | 41 | 3.2492 | 0.0012 |
| R Cerebellum | 32 -64 -54 | 40 | 2.8955 | 0.0038 |
| R Cerebellum | 42 -54 -50 | 25 | 2.8283 | 0.0047 |
| **Negative Saliences** |  |  |  |  |
| L Inferior Temporal Gyrus | -42 -56 -4 | 4706 | -13.9153 | <0.0001 |
| L Postcentral Gyrus and L Supramarginal Gyrus | -36 -24 30 | 6722 | -9.6801 | <0.0001 |
| R Frontal Pole | 40 40 4 | 879 | -8.1851 | <0.0001 |
| R Supramarginal Gyrus and R Postcentral Gyrus | 42 -40 34 | 6414 | -8.0279 | <0.0001 |
| R Temporal Fusiform Cortex | 38 -8 -40 | 4711 | -7.2526 | <0.0001 |
| L Frontal Pole | -26 52 -14 | 216 | -5.0669 | <0.0001 |
| L Frontal Orbital Cortex | -18 30 -16 | 54 | -3.9971 | 0.0001 |
| R Middle Frontal Gyrus | 34 32 30 | 30 | -3.1267 | 0.0018 |

Abbreviations: Montreal Neurological Institute (MNI), bootstrap ratio (BSR), right (R), left (L).
