# Supplementary material for: Foundations of Human Consciousness: Imaging the Twilight Zone
Source: J Neurosci. 2021 Feb 24;41(8):1769–78. doi: 10.1523/JNEUROSCI.0775-20.2020 (PMC8115882; doi:10.1523/JNEUROSCI.0775-20.2020)
Supplement: Extended Data Figure 3-5 — Supplementary Figure 3-5. Download Figure 3-5, DOCX file [file ns-JN-RM-0775-20-s05.docx]

**Figure 3-5.** Brain regions with statistically significant differences in relative regional cerebral blood flow between connected and disconnected states of consciousness during constant dexmedetomidine infusion revealed by Partial least square software.

| **Cluster Brain Regions** | **Peak Voxel MNI Coordinates (x,y,z)** | **Cluster Size (voxels)** | **BSR** | **p-value** |
| --- | --- | --- | --- | --- |
| **Negative Saliences** |  |  |  |  |
| R Occipital Pole | 22 -92 -22 | 1190 | -8.1937 | <0.0001 |
| R Putamen and R Thalamus | 28 -12 -4 | 3742 | -7.9028 | <0.0001 |
| L/R Ventromedial Prefrontal Cortex and L/R Anterior Cingulate Gyrus | -4 52 -16 | 1854 | -7.7759 | <0.0001 |
| R/L Posterior Cingulate Gyrus and R/L Precuneus | 6 -54 28 | 898 | -6.1803 | <0.0001 |
| R/L Anterior Cingulate Gyrus | 4 20 30 | 432 | -6.0793 | <0.0001 |
| L Angular Gyrus | -42 -60 60 | 139 | -5.8796 | <0.0001 |
| R Angular Gyrus and R Lateral Occipital Cortex | 54 -74 12 | 667 | -5.4645 | <0.0001 |
| R Supramarginal Gyrus | 64 -42 14 | 439 | -5.4346 | <0.0001 |
| L Angular Gyrus | -46 -82 30 | 763 | -5.2324 | <0.0001 |
| L Occipital Fusiform Gyrus | -30 -78 -20 | 497 | -5.0756 | <0.0001 |
| L Planum Temporale | -62 -30 14 | 279 | -4.8115 | <0.0001 |
| R Cerebellum | 8 -80 -42 | 183 | -4.729 | <0.0001 |
| R Inferior Temporal Gyrus | 52 -66 -22 | 113 | -4.6574 | <0.0001 |
| L Lateral Occipital Cortex | -46 -86 -8 | 79 | -4.4508 | <0.0001 |
| L Insular Cortex | -44 8 -6 | 166 | -4.3288 | <0.0001 |
| L Superior Frontal Gyrus | -2 50 48 | 46 | -3.9827 | 0.0001 |
| L Frontal Pole | -26 50 40 | 34 | -3.9026 | 0.0001 |
| L Amygdala | -24 -4 -12 | 113 | -3.8864 | 0.0001 |
| L Insular Cortex | -36 -16 6 | 97 | -3.6075 | 0.0003 |
| R Cerebellum | 4 -56 -44 | 108 | -3.5748 | 0.0004 |
| R Cerebellum | 38 -64 -40 | 84 | -3.5532 | 0.0004 |
| R Temporal Pole | 58 10 -24 | 20 | -3.3452 | 0.0008 |
| Brainstem / Pons | 10 -30 -32 | 43 | -3.2431 | 0.0012 |
| L Putamen | -26 0 4 | 65 | -3.2035 | 0.0014 |
| R Central Opercular Cortex | 52 -14 12 | 24 | -3.0176 | 0.0025 |
| R Cerebellum | 38 -56 -26 | 30 | -2.9457 | 0.0032 |
| L Angular Gyrus | -64 -52 22 | 29 | -2.8216 | 0.0048 |
| **Positive Saliences** |  |  |  |  |
| L Precentral Gyrus and L Postcentral Gyrus | -24 0 46 | 8615 | 9.5692 | <0.0001 |
| R Precentral Gyrus and R Postcentral Gyrus | 26 -20 52 | 7352 | 8.04 | <0.0001 |
| R Frontal Pole | 30 42 -4 | 1347 | 6.3226 | <0.0001 |
| L Inferior Temporal Gyrus | -50 -56 -4 | 466 | 4.7182 | <0.0001 |
| R Anterior Fusiform Cortex | 40 -4 -34 | 254 | 4.4719 | <0.0001 |
| L Inferior Temporal Gyrus | -48 -18 -22 | 113 | 4.0154 | 0.0001 |
| L Temporal Pole | -26 6 -48 | 82 | 3.7854 | 0.0002 |
| R Precentral Gyrus | 64 6 14 | 48 | 3.4529 | 0.0006 |
| L Parahippocampal Gyrus | -30 -6 -32 | 73 | 3.2942 | 0.001 |
| R Supplementary Motor Cortex | 8 -6 62 | 26 | 3.0942 | 0.002 |
| R Inferior Temporal Gyrus | 60 -34 -24 | 54 | 3.0771 | 0.0021 |

Abbreviations: Montreal Neurological Institute (MNI), bootstrap ratio (BSR), right (R), left (L).

**Figure 3-6.** Brain regions with statistically significant differences in relative regional cerebral blood flow between sleep-deprived wakefulness and N2 sleep revealed by Partial least squares software.

| **Cluster Brain Regions** | **Peak Voxel MNI Coordinates (x,y,z)** | **Cluster Size (voxels)** | **BSR** | **p-value** |
| --- | --- | --- | --- | --- |
| **Negative Saliences** |  |  |  |  |
| L Cerebellum | -38, -62, -59 | 30295 | -15.0006 | <0.0001 |
| R Cerebellum | 20, -66, -59 | 627 | -14.8844 | <0.0001 |
| R Subcallosal Cortex, R/L Anterior Cingulate Gyrus, R/L Thalamus, R Posterior Cingulate Gyrus, R/L Nucleus Accumbens, R/L Putamen, R/L Caudate | 5, 17, -5 | 30483 | -14.72 | <0.0001 |
| L Frontal Pole | -36, 56, 23 | 2231 | -7.7886 | <0.0001 |
| R Superior Frontal Gyrus | 23, 17, 69 | 681 | -5.5785 | <0.0001 |
| L Frontal Orbital Cortex | -26, 42, -21 | 824 | -5.5622 | <0.0001 |
| L Middle Frontal Gyrus | -54, 20, 39 | 139 | -5.4323 | <0.0001 |
| L Inferior Frontal Gyrus, Pars Triangularis | -57, 24, 5 | 128 | -5.0243 | <0.0001 |
| R Supramarginal Gyrus | 63, -30, 32 | 766 | -5.0088 | <0.0001 |
| L Supramarginal Gyrus and L Angular Gyrus | -63, -42, 41 | 270 | -4.9357 | <0.0001 |
| R Angular Gyrus | 47, -77, 38 | 44 | -4.8004 | <0.0001 |
| L Frontal Pole | -18, 42, 45 | 388 | -4.7282 | <0.0001 |
| L Middle Frontal Gyrus | -33, 23, 60 | 66 | -4.4912 | <0.0001 |
| L Superior Frontal Gyrus | -15, 23, 65 | 225 | -3.972 | 0.0001 |
| L Precuneus | -9, -69, 36 | 69 | -3.7136 | 0.0002 |
| Brainstem | 3, -44, -51 | 223 | -3.4709 | 0.0005 |
| L Precentral Gyrus | -63, 3, 30 | 14 | -3.1232 | 0.0018 |
| L Paracingulate Gyrus | -14, 15, 39 | 28 | -2.9672 | 0.003 |
| R Insular Cortex | 41, 14, -2 | 125 | -2.9588 | 0.0031 |
| L Frontal Pole | -51, 38, -17 | 11 | -2.8594 | 0.0042 |
| R Superior Frontal Gyrus | 2, 36, 60 | 27 | -2.8415 | 0.0045 |
| R Middle Frontal Gyrus | 42, 17, 36 | 19 | -2.7242 | 0.0064 |
| **Positive Saliences** |  |  |  |  |
| L Temporal Pole and L Parahippocampal Gyrus | -32, 12, -38 | 12574 | 20.6087 | <0.0001 |
| L Superior and Middle Temporal Gyrus, L Temporal Fusiform Cortex and L Heschl's Gyrus | -50, -35, 2 | 15411 | 12.1858 | <0.0001 |
| L Frontal Orbital Cortex | -35, 38, -3 | 474 | 11.9714 | <0.0001 |
| R/L Precuneus, R/L Postcentral Gyrus and R/L Superior Parietal Lobe | 2, -47, 68 | 12243 | 11.4094 | <0.0001 |
| R Middle and Superior Temporal Gyrus | 65, -11, -21 | 6008 | 9.384 | <0.0001 |
| R Temporal Pole and R Parahippocampal Gyrus | 29, 6, -38 | 1206 | 8.8947 | <0.0001 |
| R/L Supplementary Motor Cortex | 11, -11, 45 | 5016 | 8.7669 | <0.0001 |
| R Lingual Gyrus and R Occipital Fusiform Gyrus | 23, -44, -9 | 4959 | 8.4071 | <0.0001 |
| L Lateral Occipital Cortex and L Intracalcarine Cortex | -38, -68, 5 | 3677 | 7.3885 | <0.0001 |
| R/L Cuneal Cortex | 8, -87, 32 | 400 | 7.2148 | <0.0001 |
| R Superior Temporal Gyrus | 63, -11, -2 | 2496 | 6.2652 | <0.0001 |
| R Occipital Pole | 11, -101, 3 | 414 | 5.8574 | <0.0001 |
| L Precentral Gyrus | -17, -2, 33 | 669 | 5.6547 | <0.0001 |
| L Lateral Occipital Cortex | -39, -68, 47 | 367 | 4.8341 | <0.0001 |
| R Frontal Pole | 3, 68, -5 | 70 | 4.36 | <0.0001 |
| R Middle Temporal Gyrus | 63, -39, -11 | 457 | 4.1201 | <0.0001 |
| R Insular Cortex | 35, -8, 18 | 52 | 3.831 | 0.0001 |
| L Superior Frontal Gyrus | -21, 17, 53 | 34 | 3.7694 | 0.0002 |
| R Precentral Gyrus | 33, -20, 63 | 438 | 3.7114 | 0.0002 |
| L Frontal Pole | -21, 56, 3 | 235 | 3.4418 | 0.0006 |
| R Lateral Occipital Cortex | 17, -65, 59 | 257 | 3.3796 | 0.0007 |
| R Lateral Occipital Cortex | 57, -69, 15 | 33 | 3.3524 | 0.0008 |
| L Precentral Gyrus | -45, 8, 26 | 47 | 3.1837 | 0.0015 |
| L Lateral Occipital Cortex | -29, -87, 39 | 13 | 3.0273 | 0.0025 |
| R Lateral Inferior Occipital Cortex | 35, -90, -3 | 120 | 3.0156 | 0.0026 |
| R Parahippocampal Gyrus | 14, -11, -20 | 13 | 2.9179 | 0.0035 |

Abbreviations: Montreal Neurological Institute (MNI), bootstrap ratio (BSR), right (R), left (L).
